# Securing circulation pharmaceutically: Antiviral stockpiling and pandemic preparedness in the European Union

**DOI:** 10.1177/0967010614530072

**Published:** 2014-10

**Authors:** Stefan Elbe, Anne Roemer-Mahler, Christopher Long

**Affiliations:** Department of International Relations, University of Sussex, UK; Department of International Relations, University of Sussex, UK; Department of International Relations, University of Sussex, UK

**Keywords:** antivirals, circulation, governmentality, pandemic preparedness, pharmaceuticalization, stockpile, Tamiflu

## Abstract

Governments in Europe and around the world amassed vast pharmaceutical stockpiles in anticipation of a potentially catastrophic influenza pandemic. Yet the comparatively ‘mild’ course of the 2009 H1N1 pandemic provoked considerable public controversy around those stockpiles, leading to questions about their cost–benefit profile and the commercial interests allegedly shaping their creation, as well as around their scientific evidence base. So, how did governments come to view pharmaceutical stockpiling as such an indispensable element of pandemic preparedness planning? What are the underlying security rationalities that rapidly rendered antivirals such a desirable option for government planners? Drawing upon an in-depth reading of Foucault’s notion of a ‘crisis of circulation’, this article argues that the rise of pharmaceutical stockpiling across Europe is integral to a governmental rationality of political rule that continuously seeks to anticipate myriad circulatory threats to the welfare of populations – including to their overall levels of health. Novel antiviral medications such as Tamiflu are such an attractive policy option because they could enable governments to rapidly modulate dangerous levels of (viral) circulation during a pandemic, albeit without disrupting all the other circulatory systems crucial for maintaining population welfare. Antiviral stockpiles, in other words, promise nothing less than a pharmaceutical securing of circulation itself.

## Introduction

Looking back over the first decade of the 21st century, we could be forgiven for thinking that Europe was besieged by an epidemic of epidemics. It was the decade in which the United Nations Security Council first discussed a health issue (HIV/AIDS) as a threat to international peace and security. It was also the decade in which European governments had to contend with the rapid international spread of a new coronavirus causing Severe Acute Respiratory Syndrome (SARS). No sooner had the threat of SARS dissipated than governments were confronted by a cascade of pandemic flu scares – from ‘bird flu’ (H5N1) and ‘swine flu’ (H1N1) through to the more recent human infections with H7N9 in China. The battery of virus alerts quickly elevated pandemic preparedness to a top-level political priority in Europe and beyond. Reflecting this increased threat perception, security agendas evolved to explicitly incorporate ‘health security’ as a crucial addition to the portfolio of European security policy – frequently ranked on a par with the threat of terrorism.

Yet all the while there was also another, and rather less obvious, ‘epidemic’ sweeping across the European continent: an epidemic of pharmaceutical stockpiling. Spurned by intense fears of an imminent H5N1 ‘bird flu’ pandemic in 2005, governments across Europe anxiously lined up at the gates of pharmaceutical companies in order to place vast orders for scarce antiviral medications such as oseltamivir (brand name Tamiflu). Between them, the national governments of Europe would expend billions of euros over the next few years amassing new antiviral stockpiles. Yet the human pandemic of H5N1 did not materialize, and many public health planners were caught off-guard when the next pandemic was eventually caused not by H5N1, but by H1N1. As it became clear that the course of that new H1N1 pandemic would not nearly match the dire predictions that had formed the basis for so many pandemic preparedness plans, an intense public backlash against the costly pharmaceutical stockpiles ensued. Do they represent reasonable value for money, given the considerable resources expended in their creation and maintenance? Was there undue commercial influence in the decision-making processes to create those stockpiles? How persuasive and transparent is the scientific evidence that they would actually work as intended in a pandemic? All of these questions, in turn, have prompted yet a third epidemic – an epidemic of detailed reviews, exhaustive audits and lengthy hearings into the evolution of pandemic preparedness planning, carried out at institutional, national and international levels. The dissection of pandemic preparedness planning is now in full swing.

Scholars of security studies have made vital contributions to that dissection, using pandemic preparedness policy to illustrate the rapid expansion of security agendas to incorporate health-based threats (Cooper, 2008; Elbe, 2003, 2009, 2010b; Enemark, 2009; Lakoff and Collier, 2008; McInness and Lee, 2006; Rushton and Youde, 2014). The new notion of global health security has also formed the basis for detailed studies into the social dynamics and political implications of securitizing international health issues (Davies, 2008; Elbe, 2006, 2010a; McInnes and Rushton, 2013). Further scholarship has attended to the play and proliferation of anticipatory logics in pandemic preparedness planning (Diprose et al., 2008; Lakoff, 2008; Whitehall, 2010), and has even explored pandemic flu as the manifestation of a new ‘preparedness’ paradigm in security policy (Anderson, 2010; Lakoff, 2008; Lakoff and Collier, 2008; Samimian-Darash, 2011, 2013; Stephenson and Jamieson, 2009).

One critical area of pandemic preparedness planning, however, has so far attracted comparatively little attention in security studies. Very few scholars have looked in detail at the material technologies that lie at the heart of the pandemic preparedness apparatus: pharmaceuticals. Novel pharmaceutical products – such as the antiviral medication Tamiflu – were widely identified by governments in Europe and around the world as the ‘first line of defence’ against pandemic threats, and as the cornerstone of 21st-century pandemic preparedness planning. The two frequently went hand-in-hand, even to the extent that they often appeared synonymous with one another. All of a sudden, pharmaceuticals have thus become quite central to security policy. So, how did governments come to view pharmaceutical stockpiling as such an indispensable element of pandemic preparedness? What are the underlying security rationalities that rapidly rendered antivirals such a desirable option for government planners?

This article locates antiviral stockpiling within the emergence of a wider governmental economy of power shaping contemporary security policy. Drawing upon an in-depth reading of Foucault’s notion of a ‘crisis of circulation’, the article shows how the rapid and widespread rise of pharmaceutical stockpiling across Europe is integral to a governmental rationality of political rule concerned with managing an array of inherently *circulatory* threats to the welfare of populations – including their health. The article illustrates how a pandemic is a quintessential example of such a ‘crisis of circulation’. A pandemic is caused by the rapid international circulation of a potentially lethal virus, and is also amplified by an array of other circulatory systems – such as the international aviation network. At the same time, a pandemic is a direct threat to all of those wider circulatory systems, because controlling the spread of the virus would lead to the drastic cessation of most, if not all, other forms of circulation – as fear takes hold and emergency public health interventions are implemented. Here, novel antiviral medications such as Tamiflu emerge as such an attractive policy option because they could mark a new way of modulating dangerous levels of (viral) circulation that – unlike vaccines – can be immediately deployed following the emergence of a new pandemic influenza virus. More than that, they form the one intervention that governments could use *without* having to disrupt all the other circulatory systems crucial for maintaining population welfare. The seductive political promise of antiviral stockpiles, in other words, is nothing less than the pharmaceutical securing of circulation itself.

## A pharmaceutical epidemic: Antiviral stockpiling across Europe

The World Health Organization (WHO, 2007: 47) warns that a new pandemic infecting roughly 25% of the world’s population (a figure derived from previous pandemics) would affect more than 1.5 billion people and cause enormous social disruption due to a rapid surge in illnesses and deaths. Even in the ‘best case’ scenario of producing only relatively mild symptoms, a pandemic would create substantial healthcare costs and require governments to implement costly pandemic management plans – both of which could weaken the prospects of a recovery in the world economy (Smith et al., 2009). Those stark warnings have turned pandemic preparedness into a pressing political priority for countries in the European Union (EU) and around the world.

The need to respond to such microbial vulnerabilities is also animating a widening of security agendas to explicitly include a number of health-based threats (European Commission, 2009; WHO, 2007). So great is the importance that governments and other actors now attach to managing acute, transnational infectious disease threats that they have coined the new notion of ‘health security’, which is now proliferating in a wide array of international policy debates and official documents (Elbe, 2009, 2010b; European Commission, 2009; European Council, 2008; Global Health Security Initiative (GHSI), 2002; World Health Assembly (WHA), 2001; WHO, 2007). The WHO (2007: ix) has even made the strengthening of health security one of its core strategic priorities for the coming years, defining it as ‘the activities required, both proactive and reactive, to minimize vulnerability to acute public health events that endanger the collective health of populations living across geographical regions and international boundaries’.

Echoing those international developments at the European level, the European Commission (2011) has similarly spent much of the past decade developing its own health security framework – focusing on the three pillars of prevention, preparedness and responses to threats. A new agreement on strengthening EU health security reached at the end of 2013 extended the existing European coordination mechanism for communicable diseases to cover *all* health threats of biological, chemical, environmental and unknown origin. It also provided an institutional foundation for the EU Health Security Committee, which had been newly established as an informal committee after the 2001 anthrax letters in the United States. The draft decision even created a new legal basis for the (voluntary) joint procurement of pandemic vaccines – which is intended to help member-states achieve lower prices and allow greater flexibility, and to create more equitable access given limited production capacities at the global level (EU, 2013).

That last element was not only a particularly complex area of diplomatic negotiation, but – more crucially – exemplifies just how central the procurement of pharmaceuticals has become for European security policy in the space of just a couple of years. It was only a few years ago – in 2005, to be exact – that many governments across Europe first rushed to amass such vast pharmaceutical stockpiles for the purposes of strengthening health security. The arrival of dead birds infected with highly pathogenic avian influenza (H5N1) at the eastern borders of the EU triggered that stockpiling frenzy, especially of antiviral medications such as Tamiflu (manufactured by Roche). As William Burns, head of Roche’s pharmaceuticals division, put it in October 2005: ‘Following four ducks (that died) in Romania last weekend, Europe went mad. I don’t think you’ll find a single pack (of Tamiflu) in Paris. And this is not because we’ve had an influenza outbreak’ (cited in Turner, 2005). The epidemic of pharmaceutical stockpiling that would rapidly sweep across Europe had begun.

The year 2005 would also witness the first sustained runs on Tamiflu in several European countries. David Reddy, similarly working for Roche at the time, recalls how ‘in one country we sold within a week the amount that we would normally sell in an entire year! We had to give priority to government orders as well as ensure treatment of people during the regular influenza season’ (cited in Samii and Van Wassenhove, 2008: 7). Even though there was still no firm evidence that H5N1 could spread efficiently between human beings (the precondition for a pandemic), governments across Europe scrambled to create sizeable stockpiles of antiviral medications in anticipation of an imminent threat. Anxious as to what may lie around the corner, governments became gripped by an almost effervescent frenzy as they now competed with one another to rapidly stockpile scarce global supplies of Tamiflu from the manufacturer. All the while, national policy planners were nervously looking over their shoulders at other member-states to see how much they were stockpiling, keen to secure early deliveries and locking in orders before limited supplies ran out.

In hindsight, the pace and scale of the drive towards large-scale pharmaceutical stockpiling across most European countries is breathtaking. After studying pandemic preparedness plans across Europe in 2007, one expert concluded that ‘EU countries have so far bought close to half of all Tamiflu doses produced globally’ (Trakatellis, 2007: 22).

**Figure 1.**
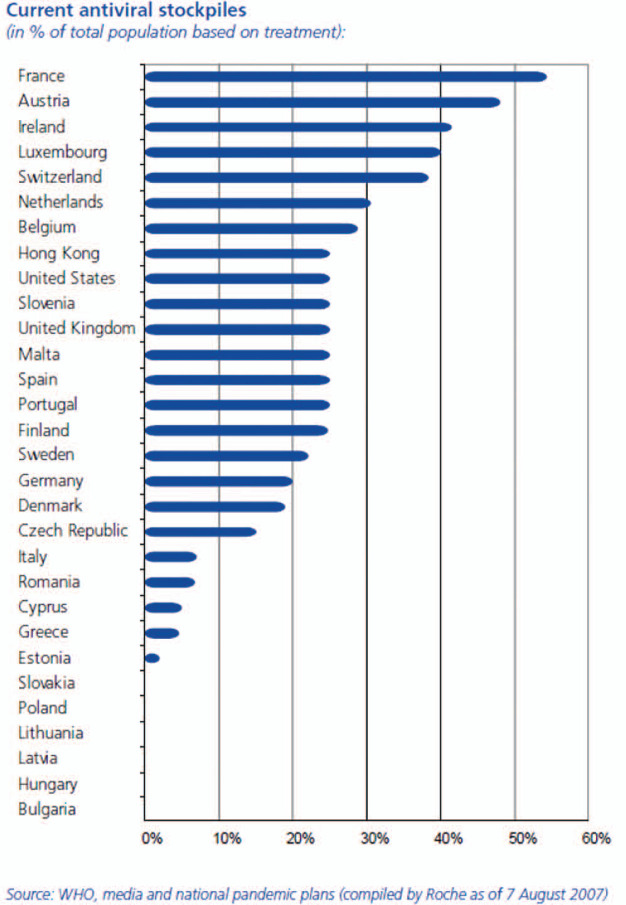
Government antiviral stockpiling levels in August 2007, as reported by Roche. *Source*: Trakatellis 2007: 23.

By 2007, countries such as France, Austria, Ireland, Luxemburg and Switzerland had reportedly set stockpiling targets in excess of 30% of the population, while countries such as the Netherlands, Belgium, Hong Kong, the United States, Slovenia, the United Kingdom, Malta, Spain, Portugal, Finland and Sweden set them in excess of 20% (Trakatellis, 2007: 23; see Figure 1). The trend towards large-scale antiviral stockpiling would continue apace during the following two years. By 2009, a total of 95 governments around the world had reportedly purchased or ordered pandemic Tamiflu stockpiles.

All in all, Roche announced, around 350 million treatment courses (3.5 billion doses) were supplied to governments worldwide between 2004 and 2009 alone (Reddy, 2010: 35–40). Although the exact price paid by governments remained confidential in most countries, the estimated cumulative costs are likely to run into billions of euros across the member-states of the EU. If there is one anticipatory security practice that materially symbolizes the rise of pandemic preparedness more than any other, it is surely this rapid surge to create vast new pharmaceutical stockpiles of antiviral medications. Indeed, the entire pandemic preparedness apparatus that has been erected over the past decade is unthinkable without the central role played by pharmaceuticals at the very heart of that structure.

The widespread move towards large-scale stockpiling of antivirals marks a novel development in European security policy in three respects. First, and as we will explore in further detail later, these antiviral medications represent an entirely new class of medicines called neuraminidase inhibitors. Although older types of antivirals were used for treating influenza infection in prior decades, the development of this new class of neuraminidase inhibitors was dependent on quite significant scientific and technological advances in virology, biochemistry and pharmacology. Neuraminidase inhibitors such as Tamiflu were only developed commercially as recently as the mid- to late 1990s, and did not receive regulatory approval in Europe until 2002. First and foremost, neuraminidase inhibitors such as Tamiflu therefore constitute a new and previously unavailable pharmaceutical intervention that governments could have at their disposal for pandemic preparedness planning. They would no longer have to rely solely on the much older vaccine technology.

Second, those antiviral stockpiles also represent a new – or at least augmented – societal deployment of pharmaceuticals. While pharmaceuticals have been routinely used in medical care for decades, the significance and function of antiviral stockpiles stretch beyond the confines of routine healthcare, trespassing deeply into the domain of national security policy. In fact, antivirals such as Tamiflu have become part of a whole new discursive category of medicines labelled ‘medical countermeasures’ – a term reserved for precisely those pharmaceuticals such as Tamiflu that exist at the intersection of health and security policy, and that can be made available to the civilian population during an emergency. The augmented security significance of those medications also goes some way towards explaining why – physically – antiviral stockpiles are often kept separate from other medicines destined for use in routine healthcare. In many European countries, the creation of these antiviral stockpiles led to the identification of novel spaces for storing them, while in some countries (including the United States) the packaging of the capsules for pandemic use was also changed to indicate their special pandemic preparedness role. In most instances, such antivirals are now stored in large, separate warehouses capable of maintaining the required environmental conditions. Those warehouses have special security arrangements in place to protect their contents in the event of a pandemic, which is also why the precise location of these warehouses remains secret in most countries. The fact that these antivirals are now deliberately acquired for broader security purposes, and with security considerations expressly in mind, marks a second novel aspect of those pharmaceutical stockpiles.

Finally, antiviral stockpiles also represent a significant development within the much longer history of strategic stockpiling. Historians trace the broader practice of stockpiling back at least 4000 years, usually on the basis of a reference in the Old Testament to Egypt building a stockpile of food equal to two years of normal consumption (National Research Council (NRC), 2008: 133). There is nothing new about stockpiling, per se. There is, to be sure, also a considerable history of stockpiling strategic resources crucial to maintaining a war effort during the Cold War (Snyder, 1966). Yet those 20th-century precedents of national stockpiling were predominantly focused on minerals and other strategic goods required for sustaining combat operations, or on keeping the economy afloat – as in the case of the creation of oil reserves in 1973 following the energy crisis of that year.

The recently established antiviral stockpiles stand out against the backdrop of this longer historical experience of stockpiling because they are devoted specifically, and even exclusively, to medicines and pharmaceuticals. They are part of a wider *biological* turn in security policy where, as Melinda Cooper (2008: 75) argues, ‘the frontier between warfare and public health, microbial life and bioterrorism [has] become strategically indifferent’. With the rise of the twin biological threats of pandemics and bioterrorism, the kinds of materials now deemed crucial to national security are not confined to those narrowly related to military efforts, or even to the broader maintenance of the economy – but also include the overall health of the population. Security policy needs ‘to arm itself against the generic microbiological threat, from wherever it might emerge’ (Cooper, 2008: 75). Pharmaceuticals are emerging as the weapon of choice.

Yet no sooner had governments begun to create those towering pharmaceutical stockpiles than the whole practice quickly became embroiled in a number of intense public controversies. Many of those controversies were triggered by the unexpectedly mild experience of the 2009 H1N1 outbreak. The 2009 H1N1 pandemic was ‘unexpectedly’ mild in the sense that the morbidity and mortality rates of the virus did not nearly mirror the ways in which a future flu pandemic had been widely predicted by a number of elaborate socio-economic models, as well as the dramatic large-scale simulation exercises in which many public officials had participated. Nor, of course, did the experience of H1N1 in 2009 and 2010 match the way in which the catastrophic experience of pandemics had been more publicly premediated in a series of popular fiction novels and blockbuster films – from *Outbreak* and *28 Days Later*, all the way through to *Contagion* (Aradau and Van Munster, 2011; De Goede, 2008). A public backlash against these antiviral stockpiles soon ensued.

Today, probing questions are being openly raised as to whether the initial expenditure on these antiviral stockpiles was ever justified in the first place (National Audit Office (NAO), 2013). Investigative journalists have expressed disquiet about whether the commercial interests of large pharmaceutical companies may have unduly influenced the political decisionmaking leading up to the creation of these stockpiles – especially in the United States government and at the WHO (Cohen and Carter, 2010; Stanton, 2005). All the while, Tamiflu has also found itself at the eye of a much larger political storm about insufficient public access to detailed clinical trial data that is used to demonstrate the efficacy and safety of new drugs in general. This latter dimension has been the subject of intensive scrutiny by groups – such as the Cochrane Collaboration – who conduct systematic reviews of the evidence base for the efficacy of drugs (Jefferson et al., 2010). In many ways, antiviral stockpiling has now become as controversial as it has been pervasive in Europe.

## Security, circulation, and governmentality

Given the enduring public controversy surrounding Tamiflu, how did governments first come to view pharmaceutical stockpiling as such an indispensable element of pandemic preparedness planning? What are the underlying political rationalities that rendered pharmaceutical stockpiling such an attractive policy response for governments across Europe? Taking a broadly genealogical perspective, at least three crucial transformations in the rationalization of government had to occur for this recent ‘epidemic’ of pharmaceutical stockpiling to unfold across Europe. Those transformations are described in Michel Foucault’s (2007) influential and well-known lecture series on the emergence of a new form of political rationality he called ‘governmentality’.

First, and again viewed in a much longer historical perspective, security policy would have to become broadly concerned with improving the welfare of populations – rather than just with the more narrow task of securing the rulers and their power. This, Foucault famously argued, is one of the key features of the new ‘governmental’ economy of power that began to emerge in Europe from the 18th century, and that rationalizes political rule precisely around a new political object of the ‘population’. The ‘population will appear above all else as the final end of government’, and it now ‘appears as the end and instrument of government rather than as the sovereign’s strength’ (Foucault, 2007: 105). From that point onwards, political rule is increasingly articulated with a view to ‘improv[ing] the condition of the population, to increas[ing] its wealth, its longevity, and its health’ (Foucault, 2007: 105). Pharmaceutical stockpiling is integral to this political rationality because it is intended – and legitimated publicly – as a way of protecting the welfare of populations. Indeed, the very reason those stockpiles are built on such a large scale is to make it possible to extend antiviral protections to the population as a whole.

Second, security policy would also have to directly encompass care for the underlying *biological* dynamics shaping population welfare. Security could not be confined to protecting and defending the territory of the state, or even organizing the material enrichment of society; it would also have to become intimately concerned with managing the complex biological processes affecting populations. Here, Foucault traced how the ‘population’ comes to be partially understood as a biological mass, the statistical analysis of which reveals that the population is constituted by ‘living beings, traversed, commanded, ruled by processes and biological laws. A population has a birth rate, a rate of mortality, a population has an age curve, a generation pyramid, a life-expectancy, a state of health, a population can perish or, on the contrary, grow’ (Foucault, [1981] 2007: 161). Designed to protect the health of populations from the biological threat of infectious disease, pharmaceutical stockpiles are integral to a political rationality that also encompasses the active management of biological dynamics underlying the population (Foucault, 1976: 142–143).

That said, there are plainly very many different diseases affecting the health of populations – most of which are dealt with through private or national systems of healthcare. Only very few, if any, of those other diseases have prompted the same large-scale creation of pharmaceutical stockpiles in the way that the threat of pandemic influenza has recently witnessed. What is it about the threat of pandemic flu in particular that necessitates such an extraordinary policy response? The intensified political problematization of *circulation* is a third genealogical transformation in the rationalization of political rule that becomes relevant here.

To illustrate this crucial dimension of governmentality, Foucault first contrasted it with the older form of sovereign power. Sovereign power largely revolved around rulers wishing to hold onto their territory and trying to conquer new territory. In that historical context, security was always a problem of the security of territory and of the sovereign who rules over the territory, trying to ensure: ‘How can it not change, or how can I advance it without it changing? How can the territory be demarcated, fixed, protected, or enlarged?’ (Foucault, 2007: 64–65). In the sovereign economy of power, security was principally concerned with pinning things down, keeping things stable, and with making sure things do *not* circulate.

The newer governmental economy of power emerging in 18th-century Europe, by contrast, sought to achieve pretty much the opposite: it tries to preserve and even incite circulation understood ‘in the very broad sense of movement, exchange, and contact, as form of dispersion and also as form of distribution’ (Foucault, 2007: 65). The art of political rule comes to consist of managing circulation and ensuring that everything remains in motion in order to maximize the prosperity and welfare of the population (Foucault, 2007: 65). Government begins ‘more or less [to] turn on the problem of circulation’ (Foucault, 2007: 64). Liberalism would emerge here as a crucial political rationality and technology for inciting such circulation – with its incessant critique of excessive government, as well as its emphasis on governing precisely by

not interfering, allowing free movement, letting things follow their course; *laisser faire, passer et aller* – basically and fundamentally means acting so that reality develops, goes its way, and follows its own course according to the laws, principles, and mechanisms of reality itself. (Foucault, 2007: 48–49)

In the era of governmentality, political rule becomes increasingly concerned with allowing, enabling, facilitating and inciting circulation.

This wider problematization of circulation would also begin to penetrate and shape security policy. At the most immediate level, one could broadly say that in the era of governmentality security policy too becomes much more intimately concerned with regulating, or sorting, various systems of circulation – the circulation of people, of weapons, of finance, of pollution, and so forth. The problem of security is now

no longer that of fixing and demarcating the territory, but of allowing circulations to take place, of controlling them, sifting the good and the bad, ensuring that things are always in movement, constantly moving around, continually going from one point to another, but in such a way that the inherent dangers of this circulation are cancelled out. (Foucault, 2007: 65)

Put more succinctly, security policy comes to be more intimately concerned with ‘how should things circulate or not circulate?’ and with sorting out the ‘good’ from the ‘bad’ circulation (Foucault, 2007: 65; see also Dillon and Lobo-Guerrero, 2008).

Yet a careful reading of Foucault’s lecture series *Security, Territory, Population* indicates that this observation really only begins to scratch the surface of the complicated relationship between circulation and security. In fact, that relationship runs much deeper than merely sorting the ‘good’ circulation from the ‘bad’ circulation (defined broadly in terms of how it impacts the welfare of the population). The transition towards a governmental economy of power will also give rise to a whole new category – or class – of security threats. For there will be specific circulatory systems that have a natural tendency to spiral out of control in a way that directly undermines population welfare. Foucault argued that such an inherently unstable system of circulation, which could not simply be left to circulate freely, begins to constitute a new kind of ‘crisis’. Indeed, a *crisis* would now come to consist precisely of any ‘phenomenon of sudden, circular bolting that can only be checked either by a higher, natural mechanism, or by an artificial intervention’ (Foucault, 2007: 61). Those new ‘crises’ of circulation are the correlative of a particular way of rationalizing political rule according to the principles of liberalism and *laisser faire*. They effectively represent the ‘dark side’ of a rationalization of political rule bent on allowing the free play of social dynamics and constantly seeking to stimulate circulation (Elbe, 2007, 2012).

With the rise of the era of governmentality, then, security policy becomes about more than just the traditional geopolitical games of territorial influence. It also becomes about managing circulation and sorting the ‘good’ from the ‘bad’ circulation. More still, it becomes concerned with identifying precisely those social phenomena that cannot be left to circulate freely lest they spiral out of control and begin to threaten the welfare of the population. Such ‘crises of circulation’ would increasingly come to find their place on the security agenda alongside more traditional concerns surrounding the deployment of armed force in the international system. Indeed, the proper art of practising security would come to consist not just of responding to those circulatory crises once they emerge, but of proactively anticipating and preparing for their emergence. Security policy would have to operate in relation to an essentially contingent and open ‘future that is not exactly controllable, not precisely measured or measurable and that … takes into account precisely what might happen’ (Foucault, 2007: 20). It will ‘try to plan a milieu in terms of events or series of events or possible elements, of series that will have to be regulated within a multivalent and transformable framework’ (Foucault, 2007: 20). As we will see next, that incessant and anticipatory problematization of circulation also lies at the heart of pandemic preparedness. For what is a pandemic if not the quintessential example of a crisis of circulation?

## Pandemics: A crisis of (viral) circulation

Pandemic threats are deeply imbricated with the problem of circulation. First, pandemic influenza is an inherently circulatory threat in that it is caused by a potentially lethal virus that first passes (in all likelihood) from animals to humans, and then circulates between human beings – each of whom may go on to infect yet more people, so enabling the virus to become epidemic and eventually pandemic. A pandemic is essentially an unpredictable and dangerous system of *viral* circulation. As Angus Nicoll, head of the influenza programme at the European Centre for Disease Prevention and Control (ECDC) puts it:

European policy-makers and politicians are put in a hard place by the prospect of modern influenza pandemics. They don’t know when one is going to happen, where it will start or what it will be like. The only certainty is that future influenza pandemics will occur and they will be unpredictable. (Nicoll and Sprenger, 2011)

A pandemic thus emerges as a system of ‘bad’ circulation directly threatening the welfare of the population by potentially causing very widespread morbidity and mortality, as well as an array of wider social, economic and political impacts.

Again, however, we are just scratching the surface of the multifaceted relationship between circulation and pandemic threats. After all, such systems of ‘bad’ circulation abound. Why have pandemic threats attracted such a particularly intense political salience in many European countries – to the point where in the United Kingdom’s National Security Strategies, for example, pandemic threats are ranked as a (top) Tier 1 threat on a par with terrorism? Pandemic flu has such deep traction as a security threat because it also sits at the very nexus and interspaces of so many other systems of circulation – of viruses, of animals and livestock, of trade, of food, of people, of children, of airplanes, and so forth. A pandemic is a system of circulation intimately connected to almost all other systems of circulation that are crucial to maintaining population welfare. Because viruses reside and replicate inside the human body, they cannot be easily separated from all those other circulations – giving influenza viruses the potential to rapidly expand around the world. Its location at the heart of so many different circulatory systems means that the virus will rapidly lead to ‘multiplying cases that multiply other cases in an unstoppable tendency or gradient’ as they begin to affect an ever-growing range of circulatory systems (Foucault, 2007: 61). Could there be a more telling contemporary example of the ‘phenomenon of sudden, circular bolting’ to which Foucault (2007: 61) referred?

Yet the relationship between circulation and pandemic threats gets more complicated still, for the perceived risk of a pandemic only increases the more all these other systems of circulation are further intensified, speeded up and extended in geographic scope – for example, through the rapid expansion of international air travel. As Gro Harlem Brundtland (2003: 417), the former Director General of the WHO, put it in an article on global health security:

today, in an interconnected world, bacteria and viruses travel almost as fast as e-mail and financial flows. Globalization has connected Bujumbura to Bombay and Bangkok to Boston. There are no health sanctuaries…. Problems halfway around the world become everyone’s problem.

Pandemics are thus often understood as the unintended ‘blowback’ – or even as the epidemiological footprint – of intensified globalization (Elbe, 2007, 2012). The reason pandemic threats strike at the very heart of a governmental rationality is because the risk of their materialization is only increased by all the government efforts to incite, intensify and extend circulation. The more circulation is intensified, the greater the risk of a new pandemic emerging – as human contacts are multiplied, animal habitats become encroached upon and movements are accelerated.

Still we have not captured the deepest threat posed by a pandemic to a governmental economy of power; for one of the most significant social effects of a pandemic, when it does emerge, is that it begins to abruptly cancel out all the other systems of circulation crucial to maintaining population welfare – such as the movement of goods, of people, of services, and so forth. Containing or at least mitigating a pandemic creates immense social and political pressure to introduce travel restrictions, the closure of schools and the cancelling of large-scale gatherings such as sports events and so forth. Even where such public health measures are resisted, recent experiences with SARS and H5N1 indicate that the fear they induce alone can have much the same effect – crippling trade, restaurant visits, public and commercial transport, and so forth as people shun public places to minimize the risk of becoming infected. Beyond the individual morbidity and mortality pandemics cause, the principal social effect of a pandemic is that it ends up inhibiting, reducing and stifling a range of other circulatory processes. Yes, a pandemic is a circulatory threat; yes, a pandemic is a system of ‘bad’ viral circulation; yes, a pandemic is fanned by a host of other circulatory systems; and yes, the emergence of a pandemic is a threat that only becomes greater the more that circulation is intensified. However, the ultimate effect of a pandemic is also that it ends up shutting down all other systems of circulation, leading to stasis. A pandemic is the quintessential ‘crisis of circulation’ because it is a circulatory threat to the very notion of circulation itself.

## Beyond vaccines: Securing circulation pharmaceutically

What can governments do to protect populations against pandemic threats? Is there, to remain with Foucault’s (2007: 61) terminology just a little bit longer, any ‘higher, natural mechanism’ or ‘artificial intervention’ that governments could adopt in order to secure their populations against the emergence of such a crisis of (viral) circulation? The traditional mechanism that Foucault himself referred to in his lecture series *Security, Territory, Population* was vaccination. Reflecting on the threat posed by smallpox in the 18th century, Foucault argued that the discovery of a vaccine meant that the problem of smallpox could now be contained through a ‘higher, natural mechanism’ – in this case, the human immune system. By exposing people in advance to small doses of the disease, the natural human immune system could develop new antibodies, allowing people to quickly fight off future infections – and before the infectious disease could take hold in the population as a whole. Of course, the introduction of vaccination during this historical period still predated the modern germ theory of disease, as well as our contemporary understanding of the workings of the human immune system. At the time, vaccination in fact stood completely apart from, and very much outside, accepted medical knowledge. It was not even known how or why the practice of vaccination worked. It was simply a matter of trial and error and empirical record that it did (Foucault, 2007: 58).

The fact that it evidently worked meant that one could now raise additional statistical questions about what chances an individual had to succumb to smallpox, or to acquire smallpox when vaccinated, and indeed how the vaccine would affect the distribution of the disease in the population, and so forth. The availability of vaccines thus gave rise to a new logic of managing infectious diseases that was not based on the sovereign principle of exclusion, as was historically the case with leprosy, where those infected were simply excluded physically from society. Nor was it the disciplinary logic of quarantine, as had been the case with plague in the Middle Ages. Instead, it was the question of efficiently managing smallpox and keeping it within socially and economically acceptable limits by stimulating a ‘higher, natural mechanism’ through vaccines to contain its circulation (Foucault, 2007: 10).

Foucault’s discussion implicitly recognizes just how desirable vaccines are to governments as a technology for managing the problem of infectious diseases. They are preventative, can have a high rate of success, and can be extended to the entire population without major material or economic difficulties (Foucault, 2007: 58). In addition – returning to the threat of pandemic flu today – we can see that vaccines also continue to remain the most desirable intervention against pandemic flu for many governments. According to the WHO (2009), ‘vaccines are among the most important medical interventions for reducing illness and deaths’ available today. In an ‘ideal’ world, many governments would thus like to acquire the capacity to routinely vaccinate their populations against the threat of pandemic influenza, and would then no longer have to worry about the destabilizing threat it poses. All kinds of flows and systems of circulation could continue to unfold unfettered.

Unfortunately, there is a major catch when it comes to vaccines for influenza. Precisely because vaccines work through the advance stimulation of the human immune system (provoking it to create new antibodies), they have to be virus-specific in order to be effective. In the case of pandemic flu this is a major problem, because influenza viruses are constantly changing and evolving. The incessant circulation of influenza viruses also fans their continuing mutation and evolution. Even vaccinating citizens for seasonal flu requires constant monitoring of the evolution of influenza viruses circulating around the world, as well as a considerable amount of educated guesswork to predict which strands of the virus are likely to be circulating in the next flu season so as to mass produce the correct type of vaccine.

This problem is exacerbated in the case of *pandemic* flu because – by definition – it is not possible to know in advance exactly what form a new virus might take. A pandemic is usually caused not by a virus that evolves gradually from season to season (genetic ‘drift’), but by one that entails a more substantial recombination of viral material (genetic ‘shift’) to which humans may have much less or even no prior immunity. This makes it extremely difficult to develop a preventative vaccine *prior* to any flu pandemic. Nor can governments simply wait for a new virus to emerge and then quickly mass produce a new vaccine. In the current model of vaccine production, it would take at least six to nine months to mass produce any new vaccine. Even countries that have their own domestic vaccine-production capabilities (and most countries in the world do not) would have to endure the effects of a pandemic for many months without the widespread availability of a vaccine for the population. Even then, there would not be enough international supply to meet global demand.

The unsavoury and thorny dilemma that pandemic flu therefore poses for governments is as follows. Either they would effectively have to choose to let the virus run its course for months while they wait for new vaccine to become gradually available – with all the wider social, economic, political and health implications that would entail – in which case governments would also risk being seen as weak and even negligent in their core duty to protect the welfare of their populations. Or, they would have to fall back on a disciplinary economy of power and implement a range of much more ‘draconian’ public health measures aimed at curtailing the movement of people in the hope of reducing human contacts – such as school closures, cancelling public events, quarantine, isolation, and so forth. Like the first option, however, that course of action would also have the effect of shutting down most systems of circulation within the population and drastically undermining its overall welfare. The interventions would not be that much more desirable than the underlying problem they are intended to address.

Antivirals are attractive to governments because they could offer a partial way out of this thorny dilemma. Antivirals were commercially developed during the late 1990s as an alternative way of managing the circulation of influenza viruses in the population. Unlike vaccines, antivirals do not stimulate the human immune system in advance of infection so that the human immune system can then neutralize new cases of infection in future. Instead, this new generation of pharmaceuticals seeks to directly interfere in a targeted way with the molecular processes of viral circulation that take place inside the human body. It is a well-known fact that viruses – including influenza viruses – cannot replicate by themselves. In order to replicate, they need to insert themselves into existing cells, and then use those cells to make more copies of themselves. The newly formed virus particles then leave the cell again, destroying the host cell in the process and going on to infect neighbouring cells – repeating the cycle over and over again (Schneider, 2001). In evolutionary terms, it is an elegant and sophisticated process, albeit one that also has a ‘catch’. As the viruses leave the host cell, they become attached to a coating of sialic acid found on the surface of the host cell. They thus require an enzyme – called neuraminidase – in order to dissolve this ‘sticky’ acid and to free themselves so that they can go on to infect further cells (Schneider, 2001). Without this enzyme, the new virus particles would simply remain stuck on the host cell with nowhere to go.

If there were a way to artificially disrupt, or inhibit, the working of this neuraminidase enzyme in the human body, it could mark an entry point for a new type of pharmaceutical intervention – a neuraminidase inhibitor. Tamiflu (oseltamivir) – and a closely related predecessor drug called Relenza (zanamivir) – are two attempts to capitalize on recent advances in virology, biochemistry and pharmacology in order to deliberately and rationally design a new, artificial pharmacological compound that would be capable of ‘blocking’ this enzyme so crucial to viral circulation. These new antivirals do not actively destroy viruses in the way that many antibiotics destroy bacteria; however, they do promise to slow the process of viral replication inside the human body, barring viruses from releasing themselves and going on to affect new cells. That is also why they must generally be taken within 48 hours after the onset of symptoms, that is, before the viral infection has multiplied too pervasively in the human body.

These antivirals represent a new type of pharmaceutical intervention for directly modulating the circulation of influenza viruses in the population. More importantly still, they promise governments the option of selectively limiting the circulation of influenza viruses in the population *without* having to resort to the imposition of more ‘draconian’ public health measures that end up inhibiting other systems of circulation. As the Chief Scientist and Head of the Office of the Chief Scientist at the ECDC Professor Johan Giesecke (2012) explained in an interview:

The classical measures for public health – isolation, quarantine, mass vaccination … there is clearly an increasing resistance in the population to these more drastic public health measures…. It would be impossible today [to do mass isolation] because people would say why, I don’t want to, is this necessary, where is your data? … The classical public health measures would be questioned much more than they were 50 years ago…. This makes medical countermeasures more important today. You cannot politically do a cordon sanitaire anymore. It would probably be impossible.^1^

Antivirals, in other words, are the one ‘artificial’ intervention that governments could potentially deploy during a new influenza pandemic without having to disrupt all the other systems of circulation crucial for population welfare – such as children going to school, business trading, people travelling, and so forth. The use of antivirals promised governments the ability to largely sidestep many of the more traditional, restrictive and unpopular public health measures, and to allow all of these wider systems to continue circulating in the event of a pandemic. The seductive political promise of antiviral stockpiles, in other words, is nothing less than the pharmaceutical securing of circulation itself. And, in the case of Tamiflu, it could be as easy as popping a pill.

## Stockpiling for preparedness: Taking Tamiflu out of circulation

As Tamiflu begun to emerge as a new and crucial ‘first line of defence’ for pandemic flu, one key question remained: Would governments be able to secure sufficient quantities of antivirals during a pandemic? Put differently, would a *laisser faire* approach lead to the correct alignment between the volumes of available and required antivirals during a pandemic, or would this too necessitate some kind of ‘artificial’ advance intervention by governments? Based on the historical experience of pandemics, most preparedness plans envisioned needing supply levels capable of treating around a quarter of the population – although some countries set targets in excess of 50% of the population. As governments drew up their pandemic preparedness plans, it became clear that a policy of *laisser faire* would not, in fact, generate the required volumes of antivirals under pandemic circumstances.

Part of the reason for a likely shortfall has to do with the political economy of antiviral production. The manufacturer of Tamiflu (Roche) repeatedly warned governments that in the event of a pandemic it was unlikely that there would be sufficient existing or spare capacity in the supply chains to make large quantities available. According to Mike McGuire, vice president of anti-infectives for Roche at the time, ‘once an outbreak occurs or a pandemic flu starts spreading, it will be impossible to meet immediate and widespread demand for Tamiflu’ (cited in Continuity Central, 2012). This factor is compounded by the fact that a pandemic would likely lead to a rapid surge in demand, as countries around the world would all seek to acquire large amounts of the medicine simultaneously.

Nor, Roche warned further, could governments simply wait for commercial production to be rapidly scaled up following the onset of a pandemic. Roche representatives briefed governments about how complex the Tamiflu production process is, that it is dangerous in parts, and that it involves a series of complicated steps. What is more, it is a pharmacological property of neuraminidase inhibitors that they must be administered within 48 hours of the onset of symptoms in order to have a significant effect. In terms of making these antivirals available to the population at large, governments and authorities would thus require not just large-scale access to the medication, but also *rapid* access to the medicine in order to make it available before it is too late. Some kind of artificial mechanism would be needed to align the correct levels of viral and antiviral circulation in the immediate aftermath of a pandemic.

In a context of limited international production capability and the extraneous demands that a pandemic would pose, the only way to guarantee such rapid access to large quantities of antiviral medications was to create a spare cache of medicines that would be kept on ‘stand-by’ for a future pandemic^2^. Governments, in short, would need to amass the desired quantities of antivirals in advance. So, the practice of pharmaceutical stockpiling was born, rapidly spreading across Europe (and beyond), not unlike an epidemic itself. It turned out – rather ironically in the end – that the only way to secure circulation pharmaceutically was by first taking a large number of antivirals *out* of circulation, deliberately confining them to those vast and highly secure warehouses that began to pop up across Europe. In the case of pandemic threats, the political art of preparedness came to revolve around determining which flows governments would have to deliberately immobilize and sacrifice in order to secure circulation at large.

## Conclusion: The pharmaceuticalization of security

Prompted by the public controversies surrounding Tamiflu, this article explored the political rationalities underpinning the rapid rise of antiviral stockpiling across Europe. Drawing upon an in-depth reading of Foucault’s notion of a ‘crisis of circulation’, it showed how pharmaceutical stockpiling was integral to a governmental rationality of political rule continuously seeking to anticipate myriad *circulatory* threats to the welfare of populations – including biological threats to their overall levels of health. Novel antiviral medications such as Tamiflu emerged as such an attractive policy option because they could allow governments to rapidly modulate dangerous levels of (viral) circulation *without* disrupting all the other circulatory systems crucial for maintaining population welfare. Antiviral stockpiles, in short, promised nothing less than a pharmaceutical securing of circulation itself.

Yet this trend towards large-scale antiviral stockpiling only represents the most public manifestation when it comes to the growing centrality that pharmaceuticals are acquiring in contemporary security policy. Beyond the antivirals discussed here, there are many further attempts by governments to develop, acquire and stockpile a range of other medical countermeasures against the threat of bioterrorism – ranging from next-generation vaccines and antibiotics through to other antivirals and anti-toxins. A whole host of new initiatives, and even entire new institutions, have been recently developed for this task. Today, the quest to secure populations is no longer effected solely through the conventional security technologies tied up with the deployment of armed force in the international system. Increasingly, it is also carried out through the proactive acquisition and stockpiling of a range of pharmaceutical products. Pharmaceutical reason is beginning to penetrate contemporary security policy much more widely.

This trend towards pharmaceutical solutions is not even confined to the field of security policy. Scholars from other disciplines, especially in sociology and anthropology, are tracking and exposing a much wider proliferation of pharmaceutical logics, imaginaries and strategies throughout different sections of society. Working with the concept of ‘pharmaceuticalization’, they observe a pronounced increase in recourse to pharmaceutical products across many different areas of social life (Clarke et al., 2010; Lakoff, 2005; Petryna et al., 2007; Whyte et al., 2002; Williams et al., 2009). The trend towards pharmaceutical stockpiling in security policy therefore needs to be situated within a much broader social context in which pharmaceuticals are being used much more widely, by more people, and for a more extensive range of conditions and afflictions (Abraham, 2010, 2011; Williams et al., 2009, 2011).

What is animating all these pharmaceuticalization processes? The drivers already identified in the wider social science literature include biomedical advances, which are enabling novel therapies to be developed. The broader medicalization of existence is undoubtedly another important driver. Similarly, more aggressive industry promotion, including direct-to-consumer advertising, can increase demand for pharmaceutical products (Abraham, 2010; Williams et al., 2011). The analysis of antiviral stockpiling carried out here suggests that, when it comes to understanding the contemporary dynamics of pharmaceuticalization, we also need to be attentive to the underlying rationalities of political rule within which pharmaceuticals are emerging as such attractive policy options for governments.

This ‘pharmaceuticalization’ of security is fascinating, in the end, because it transforms our bodies into crucial sites of security policy. Of course, in one way or another, our bodies have always been central sites for security practice – at least for as long as people have been fighting wars and waging battles. Yet there is also something deeper unfolding in this pharmaceuticalization of security, something that goes beyond Foucault’s own distinction between the anatamo (or disciplinary) politics of the human body and the biopolitics of the population. The pharmaceuticalization of security pushes security policy much further into the interstices of the corporal body, descending to the more minute level of the complex immunological systems driving our biological existence. The pharmaceuticalization of security is transforming the inner molecular workings of all of our bodies – and not just the bodies of soldiers – into the new battlefields of security policy. Or, to put it more succinctly, it is beginning to turn the subject of security into a patient.

## Notes

1. Interview with the Chief Scientist and Head of the Office of the Chief Scientist, Professor Johan Giesecke, at the European Center for Disease Prevention and Control, Stockholm, 30 November 2012.

2. Baker M (2010) *Quality Assuring the NPFS . . . and Further RCGP Reflections on Pandemic H1N1 (2009)*. A report on the NPFS and the H1N1 swine flu outbreak for the Chief Medical Officer. Personal correspondence.
